# Biologic Basis of De-Epithelialized Transverse Platysma Flap for Oral Cavity Reconstruction 

**DOI:** 10.22038/ijorl.2019.26361.1861

**Published:** 2019-11

**Authors:** Saeedeh khajehahmadi, Amin Rahpeyma

**Affiliations:** 1 Dental Research Center, Mashhad University of Medical Sciences, Mashhad, Iran.; 2 Oral and Maxillofacial Diseases Research Center, Mashhad University of Medical Sciences, Mashhad, Iran.

**Keywords:** Epithelialization, Platysma flap, Wound healing, Reconstruction

## Abstract

**Introduction:**

The inferiorly and laterally based platysma myocutaneous flap contains hair in some ethnics; therefore, it is required to change the myocutaneous flap to myofascial flap to prevent the hair growth after its transfer to the oral cavity.

**Materials and Methods:**

Five male mongrel dogs were selected for this study. De-epithelialized laterally based platysma flap, muscle part facing the oral cavity, was used for buccal reconstruction. The clinical healing process was photographed every week. After 40 days, biopsy specimens were obtained from the transferred flap.

**Results:**

According to the results, all flaps survived. At the end of the first week, the flap was covered with fibrinous exudate. On the third week, only the center of the transferred flap was not covered with mucosa. Within 40 days, the flap was distinguishable clinically from the adjacent buccal mucosa just by hypopigmentation. Hematoxylin and eosin staining of the biopsy specimens taken on day 40 showed thin stratified squamous epithelium covered with a tiny parakeratin layer.

**Conclusion:**

Myofascial platysma flap, muscle part faced oral cavity, survives and undergoes mucosalization after adaptation to the recipient oral tissue.

## Introduction

The platysma flap is thin and pliable; therefore, it is suitable for intraoral reconstruction (1). Superiorly based platysma flap includes the skin overlying clavicle and is useful to reconstruct the oral cavity (2). The skin in this region contains thin sparse hair follicles inducing no problem in oral cavity reconstruction. Myocutaneous inferiorly- and laterally-based platysma flap contains hair in some ethnics; therefore, it should be changed to myofascial variant in order to prevent the hair growth after being transferred to the oral cavity (3). Platysma myofascial flap is used for pharynx, laryngeal, esophagus, and oral cavity reconstruction as a cover flap (4-6). This flap can prevent Frey syndrome and depressive deformities after parotidectomy if used as interposition flap (7). Histologic changes after myofascial platysma flap, muscle part facing the oral cavity, have not evaluated yet.

## Materials and Methods

*Preparation*

Five healthy male mongrel dogs aged 1.5-3 years and weighing 17-24 kg were selected for this study. This animal study was approved by the Ethics Committee of Mashhad University of Medical Sciences, Mashhad, Iran (No: 940691). Clinical and paraclinical examinations were performed to confirm the health status of the dogs. During the study, the dogs had free access to food and healthy water. Fourteen days before the initiation of this experimental study, the animals had time to adapt to their cage and diet. 

They were subjected to vaccination and medication for parasitic infections. In this regard, each animal was premedicated with an intramuscular injection of 2 mg/kg xylazine hydrochloride (xylazine 2%, Alfasan International BV, Woerden, the Netherlands). General anesthesia was begun with ketamine (10 mg/kg) and diazepam (0.5 mg/kg) and maintained throughout the surgery.

**Surgical technique**

Under general anesthesia and after prep and drape, the skin below the inferior mandibular border was shaved. The skin island flap with the dimensions of 2×5 cm was designed in the submental region while the leading edge of the given flap was extended to the midline. Supraplatysmal dissection was performed with a sharp surgical blade from the flap leading edge posteriorly. The flap was elevated from medial to lateral direction, below the platysma muscle and underlying the fascia. 

After flap harvesting, the skin was removed in the subdermal tissue, beneath the hair follicles by means of a sharp scalpel, and finally, the donor site was sutured. The flap was brought to the oral cavity by tunneling and adapted to the intentionally created mucosal defect (deep to the buccinator muscle) in the buccal region while the platysma muscle left bare and exposed in the oral cavity.

 Immediately after the surgery, the animals were placed on a pureed food diet for 2 weeks. The clinical healing process was photographed every week. After 40 days, biopsy specimens were obtained from the transferred flap, and then fixed in 10% formalin. The specimens were processed for routine hematoxylin and eosin staining. Incisional biopsy technique was used to prevent the need to sacrifice the animals. Five dogs were chosen to provide an adequate amount of tissue. It should be mentioned that phylogenetically, platysma muscle in human is equivalent to the panniculus carnosus muscle in dogs. For simplicity, the platysma muscle term was used interchangeably in this article.

## Results

*Clinical assessment*

All flaps survived, and no infection or dehiscence was observed in the clinical examination of the donor skin. Possible surgical complications are flap necrosis and orocutaneous fistula. 

**Fig 1:**
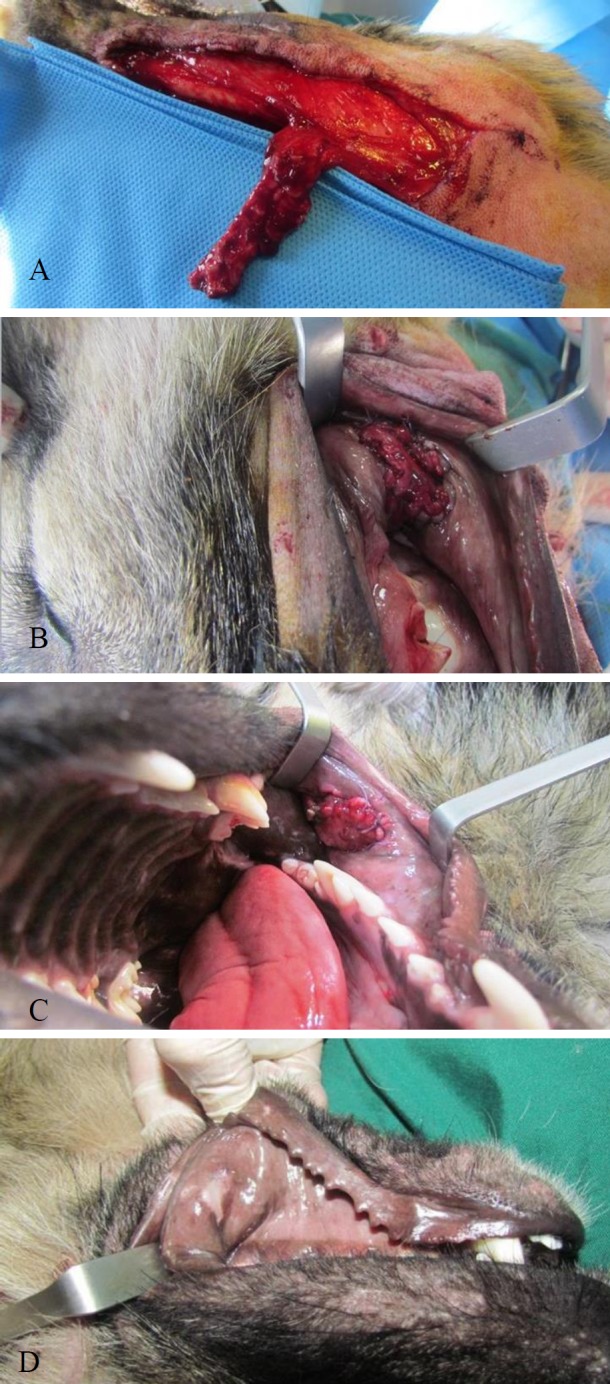
**a**) De-epithelialized platysma flap, **b**) flap adaptation to recipient bed, muscle part facing the oral cavity, **c**) a photograph taken a week after operation, **d**) presentation of hypopigmentation 40 days postoperation

Within the first week, the flap was covered with fibrin exudate, and then by fleshy tissue during the next two weeks. At the end of the third week, only the center of the transferred flap was not covered with mucosa. In addition, 40 days postoperatively, the surface of the flap was covered with mucosa but could be distinguished from the adjacent intact buccal mucosa because of its color difference (Fig.1). There was no clinical sign of fibrosis in the buccal pouch. The results were similar for all five samples.

**Histologic assessment**

**Fig 2:**
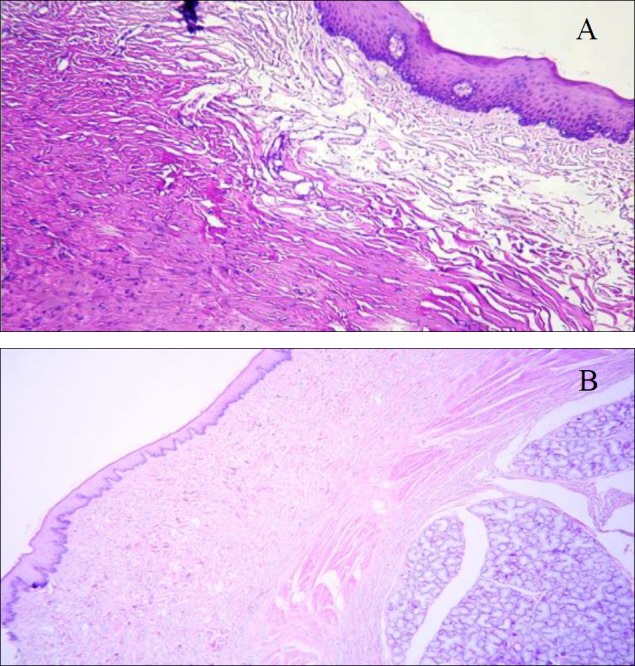
a) Thin parakeratinized epithelium with small rete pegs covering the flap surface (Platysma muscle fibers are present beneath the epithelium; H-E ×200), b) histologic view of the buccal mucosa of the dog (Parakeratotic epithelium, broad rete pegs, buccinator muscle and minor salivary glands; H-E ×40)

Hematoxylin and eosin staining of the biopsy specimens taken on day 40 showed thin stratified squamous epithelium, covered with a thin parakeratin layer. The boundary between the epithelialized flap and surrounding buccal mucosa was not distinguishable microscopically in the epithelial component (Fig.2). The platysma muscle in the flap side and minor salivary glands, lateral to the buccinator muscle in the adjacent buccal mucosa, were two distinguishing histologic landmarks in the connective tissue.

## Discussion

Myocutaneous flaps replace the lost oral mucosa with the skin. It is not an ideal reconstruction because it does not replace like with like; however, it is an acceptable method (8). Nonetheless, if the hairs are transferred to the oral cavity, they bother the patient; therefore, this issue should be given special attention. Laser epilation and second surgery to remove the hair are two solutions for this problem; however, there is also a preventive method, namely the de-epithelization of myocutaneous flap (9,10).

Pedicled muscle flaps, including temporal, sternomastoid, masseter, and free muscle flaps (e.g., serratus anterior and latismus dorsi), and myofascial pedicled flap (e.g., temporoparietal, radial forearm, and dorsal thoracic fascia free flaps) undergo secondary epithelialization when exposed to the oral cavity environment but with different contracture patterns. The naked muscle flaps are prone to severe contracture; however, myofascial flaps are healed with less wound contraction (11,12). Myocutaneous flaps are commonly used for oral cavity reconstruction. The skin is not mandatory for the survival of myocutaneous flaps (13). Skin exclusion is mainly performed for removing the hairs. Nevertheless, other reasons accounting for this measure are debulking, eliminating black pigment, reducing complication rate at the donor sites, dead space filling, and correcting contour (14-17).

Generally, in the oral cavity, de-epithelialized flaps undergo three nonseparated steps, namely inflammation, granulation tissue formation, and epithelialization, before complete epithelialization (18). Hair problems are of utmost importance in the flaps that use the skin from the submentum in the ethnics with heavy beards. This region is the skin paddle of submental and laterally (transverse) based platysma flaps.

Other myocutaneous flaps, such as pectoralis major, deltopectoral, sternocleidomastoid, and infrahyoid, include hairless skin or skin with sparse hair for intraoral reconstruction. Prevention of hair growth in platysma flaps was attempted by dermatome in previous studies. However, this technique was not effective in the prevention of hair growth (19,20). This failure can be explained by the fact that hair follicle cells are still transferred by this myodermal flap. 

Full-thickness de-epithelialization of the flap by a scalpel could solve this problem as shown in this experiment. Platysma is a thin muscle located in the outer part of the superficial layer of the cervical fascia. If during the flap de-epithelialization of the platysma myocutaneous flap, this muscle is exposed, then myofascial flap is an appropriate term. On the other hand, if the dermal and fatty tissue remains over the platysma muscle after de-epithelialization, the myodermal flap is a more accurate term. 

It is obvious that myodermal platysma flap is a little bit thicker than platysma myofascial flap. It is recommended that the fascial surface of the myofascial flaps be exposed to the oral cavity since it is believed that fascial surface protects the underlying muscle against infection (21). Platysma flaps have a random-pattern blood supply and if insisting on this rule (fascial surface up) needs to flap base twisting or torsion, it should be ignored, and the muscle surface could be sutured to mucosal edges to prevent pedicle twisting. It is anticipated that after deep de-epithelialization, hair follicles do not transfer to the oral cavity with flap transposition. 

Surgeon cannot be sure that the plan of dissection is uniform and under the hair follicles during the de-epithelialization process and some hair follicle regrowth is anticipated. If these hair shafts face the recipient bed, then infection is the result. For this reason, the authors of this article preferred platysma myofascial flap for the oral cavity while the muscle part is prepared for mucosalization. In this case, the hair regrowth can be managed easily.

Angiographic studies have shown that platysma flaps are myofascial rather than myocutaneous (22). The present study facilitated the explanation of the biologic basis of this flap, when used for oral cavity reconstruction after de-epithelialization. Hypopigmentation of the reconstructed buccal mucosa in this study comes from the fact that the migration of epithelial basal cells over the granulated myofascial flap is the mechanism of mucosalization and occurs early, while melanocyte migration is a late event (23). 

Another lesson learned from this study was that it is not necessary to cover this flap with mucosa, skin grafts, or membranes because de-epithelialized surface acts as a biologic guide for epithelial migration after granulation tissue formation. 

## Conclusion 

As the findings indicated, the myofascial platysma flap, muscle part faced the oral cavity, survives and undergoes mucosalization after adaptation to the recipient oral tissue.
